# Affective Instability and Emotion Dysregulation as a Social Impairment

**DOI:** 10.3389/fpsyg.2022.666016

**Published:** 2022-04-15

**Authors:** Philipp Schmidt

**Affiliations:** ^1^Department of Philosophy, Technical University Darmstadt, Darmstadt, Germany; ^2^Department of Philosophy, Julius Maximilian University of Würzburg, Würzburg, Germany; ^3^Phenomenological Psychopathology and Psychotherapy, Psychiatric Clinic, Heidelberg University, Heidelberg, Germany

**Keywords:** borderline personality disorder (BPD), phenomenology, social experience, affective instability, interaffectivity, empathy, emotion dysregulation, emptiness

## Abstract

Borderline personality disorder is a complex psychopathological phenomenon. It is usually thought to consist in a vast instability of different aspects that are central to our experience of the world, and to manifest as “a pervasive pattern of instability of interpersonal relationships, self-image, and affects, and marked impulsivity” [American Psychiatric Association (APA), 2013, p. 663]. Typically, of the instability triad—instability in (1) self, (2) affect and emotion, and (3) interpersonal relationships—only the first two are described, examined, and conceptualized from an experiential point of view. In this context, disorders of self have often motivated analyses of self-experience and the sense of self, affective disorders have been frequently considered in the light of emotional experience and its phenomenological structure. Patterns in the phenomenology of social experience have found comparatively little traction when it comes to the conceptualization of the interpersonal disturbances in borderline. In this paper, I argue that interpersonal instability in borderline consists in much more than fragile and shifting relationships but, most importantly, also involves certain styles in experiencing others. These styles, I suggest, may play an explanatory role for the borderline-typical patterns of interpersonal turmoil and so deserve more attention. To better describe and understand these styles, I explore the phenomenological structure of borderline affective instability and discuss the implications it might have for how a person experiences and relates to other people. Considering core aspects of borderline affective instability, such as alexithymia, emotional contagion, emotion dysregulation, and chronic emptiness, I propose borderline can be interpreted as a disturbance of interaffective exchange, which gives rise to certain ways of experiencing others that imply a social impairment.

## Introduction

Being with other people shapes most if not all aspects of our lives and determines how we find ourselves in the world. Interpersonal problems thus can affect us in a deep and comprehensive way. They can concern our lives as a whole. Interpersonal problems often form a central aspect in the lives of those diagnosed with borderline personality disorder (BPD). In BPD, these problems can consist in severe and recurring conflicts, fluctuating relationships with sudden painful disruptions, and constant tension or turmoil with significant others. Together with instabilities in one’s sense of self and affective experience, instabilities in interpersonal relationships constitute a triad that is typically recognized as being essential to BPD [Meares et al., 2011; American Psychiatric Association (APA), 2013; Tomko et al., 2014; Southward and Cheavens, 2018].

Interpersonal problems in the context of BPD are well-documented empirically (e.g., Zeigler-Hill and Abraham, 2006; Stepp et al., 2011; Hepp et al., 2018) and their various facets and factors have been investigated in many studies (Gunderson et al., 2018). Some of the salient issues in this regard are insecure attachment (Levy et al., 2011; Miljkovitch et al., 2018), hypermentalization (Sharp and Vanwoerden, 2015; Somma et al., 2019), and heightened rejection sensitivity (De Panfilis et al., 2015). Links between interpersonal difficulties and instabilities in affective experience have also been demonstrated. Hepp et al. (2018), for instance, have reported a relationship between negative affect and interpersonal problems. Other studies have emphasized the close connection between interpersonal difficulties and emotion dysregulation (Kosson et al., 2015; Euler et al., 2021; Haliczer et al., 2021), supporting the idea that emotion regulation is, at its base, socially structured (López-Pérez et al., 2016; Barthel et al., 2018). It has also been suggested that difficulty in recognizing the emotions of others is associated not only with emotion processing but also with interpersonal problems (Niedtfeld et al., 2017).

Although efforts are being made to elucidate and conceptualize the recurring and pervasive interpersonal problems associated with BPD, the *experiential* dimension of interpersonal problems has been little addressed. How do people suffering from BPD experience other people? Existing studies focus on what kind of feelings are involved in social interactions, or quantitative aspects such as the number of interaction partners (Stepp et al., 2009). Some have investigated relationship patterns (Drapeau et al., 2012), but without addressing specifically how these patterns manifest in a person’s lived experience. More structural aspects of interpersonal experience in those with BPD are likewise under-researched. However, this is an important issue, given that the experience of others not only forms part of the concrete lived experience of persons with BPD and their existential condition, but likely is also a factor in the genesis, continuation, and solidification of interpersonal issues. It is plausible that the way a person experiences others plays a role in how they socially interact, for it shapes the structure of their interpersonal relationships and determines the kinds of relationship that emerge.

The aim of this paper is to examine the structures and styles of interpersonal experience in BPD that may underlie or correlate with interpersonal problems. Two methodological choices guide the analysis: (1) I will look at interpersonal experience through the lens of phenomenological psychopathology, sharing the assumption that phenomenologically describing experience cannot only help to *understand* but also—at least to some degree—to *explain* experiential phenomena; (2) more specifically, I will look at typical aspects of affective experiences in BPD, assuming that the way a person’s emotional experience is phenomenologically structured and processed may affect how they relate to others. Before further explaining this methodological approach and clarifying the task, let me specify the research questions that will guide the analysis.

## Research Questions

There is no single universal way we experience the world, ourselves, and others. People vary significantly in how they relate to others and what kind of relationships they have. In the case of BPD, however, there seem to be typical patterns of social problems and interpersonal configurations found in quite different individuals with distinct and unique life stories. This motivates the view that, despite individual backgrounds and contexts, there are shared ways of experiencing others among those who suffer from BPD. Given the great importance that others have for all of us, recurring interpersonal problems have a major influence on the level of suffering in borderline patients. Their suffering is aggravated by the fact that often much of their desires, feelings, and cognitions center on attachment issues and connections to other people. Other people are of crucial significance to those whose lives are characterized by BPD, yet they often struggle in maintaining stable relationships and undergo painful experiences in their interactions with others. Although interpersonal problems are certainly not reducible to experiential issues, the question arises as to what role interpersonal experience plays in the genesis and processing of them. To address this question, we need to understand how individuals with BPD experience other people. This presents a significant challenge, as there is no single and general way in dealing with others; accordingly, the task is to identify as many aspects as possible that are typical to interpersonal experience in BPD. Thus, the first and guiding research question, presenting the overall framework is: *What are the characteristics or styles of interpersonal experience in BPD*?

It has often been proposed that instabilities in emotional experience are at the core of BPD (Linehan, 1993; Selby and Joiner, 2009). This suggests that instabilities in self-related perceptions and social relationships are consequences of affective disorders. While such a unidirectional etiology might be controversial and ultimately hard to defend, it nonetheless gestures toward the importance of emotional experience and its processing for how one experiences oneself and others. Struggles with one’s own emotions have a significant impact on how other people feature in one’s perception of one’s situation and environment. When one is undergoing uncontrollable anger oneself, it is difficult and sometimes even impossible to devote oneself to the emotional feelings of others, for instance, to caress and calm down a young child. Feeling overwhelmed by one’s own emotions can make it hard to empathize with others and attend to their feelings. What is correct of particular emotions also applies to affective phenomena more generally, motivating the following question: How do the well-documented and important affective phenomena in BPD such as alexithymia and difficulties in empathy (New et al., 2012), emotional contagion (Niedtfeld, 2017), emotion dysregulation (Glenn and Klonsky, 2009), chronic emptiness (Miller et al., 2020), and mental pain (Fertuck et al., 2016) shape a person’s experience of other people? The second research question is thus: *How do affective phenomena present aspects or styles of interpersonal experience in BPD?*

## Methodology

To distinguish the central aspects and styles of interpersonal experience in BPD that result from struggles with deep and traumatic emotional experiences and their regulation, I will look at borderline-specific affective phenomena through the lens of phenomenological psychopathology. The primary aim of phenomenological psychopathology is to reveal the essential structural aspects of different experiential and existential conditions. The rationale behind the approach is to reconstruct the lifeworld and its phenomenological characteristics in order to *understand* how a person experiences the world, themselves, and others. Concrete situations can be experienced quite differently by different individuals, depending on their particular situation and existential condition. Shedding light on a person’s existential condition is therefore a useful way of clarifying their sufferings and the motivations that may shape and trigger the specific behaviors that are likely to cause or aggravate problems a person has in their interpersonal relations.

Accordingly, while the focus of phenomenological psychopathology is on *description*, this can also play an *explanatory* role. Describing, and thus understanding an existential condition can help us to see how and why a person is suffering. Pioneers in phenomenological psychopathology, such as Karl Jaspers (1883–1969), emphasized its descriptive task and even denied that it is possible to provide explanations, given that he took the connections—and causal mechanisms—between different experiences to be non-conscious (*außerbewußt*, Jaspers, 1973, p. 253). For Jaspers, it is not possible to explain experiences by referring to experiences (cf. Schmidt, 2018). For instance, even if one could make sense of how an individual might end up showing general suspiciousness toward others after being badly cheated (Jaspers, 1973, p. 255), Jaspers took such connections between experiences to be at best “probable” (Jaspers, 1973, p. 260).

By contrast, contemporary researchers have stressed that phenomenological psychopathology can do more and does more than simply describing a person’s experience under some given circumstances (Fuchs et al., 2019). For there is not merely an explanatory link between how a person experiences and how they deal with the world; there are also interrelations between different experiences, as well as, notably, experiential dimensions that are structured in a law-like manner (Schmidt, 2018). Sass (2010, 2014) distinguishes at least six different kinds of relationships or connections that hold between different experiential phenomena. For instance, hyperreflexivity in schizophrenia can be seen as an attempt to compensate for a diminished sense of self. On this view, a diminished sense of self and a tendency toward reflective engagement are not two independent aspects of schizophrenic experience, but stand in a close motivational relationship: schizophrenics hyperreflect *because* they have a diminished sense of self, and hyperreflexivity just *is* a manifestation of the latter. In a similar vein, Gallagher and his colleagues (Gallagher, 2013; Gallagher and Daly, 2018), in their *Pattern Theory of Self*, suggest that in order to understand a certain condition we must investigate how the different experiential dimensions co-vary and form the specific experiential pattern that is associated with or constitutes a given existential condition. The rationale behind this is that all the different experiential dimensions typically described by phenomenological philosophy—self-experience, temporality, intentionality, embodiment, affectivity, narrativity, normativity, intersubjectivity, etc.,—together form and shape an individual’s experience of the world. These dimensions cannot be separated from each other: changes in how I am bodily situated in the world, for instance, can have an impact on my experience of others, of normativity, or of time.

Applying this approach to interpersonal experience in BPD and focusing on how the affective dimension shapes styles of social encounters and relatedness, the task then is to unravel what the affective phenomena in BPD mean for experiencing others. Crucially, relationships between the affective and the social need not be immediate and direct. That is, it may be that certain affective phenomena primarily impact on a person’s embodiment or attentional tendencies but have only secondary ramifications for the experience of other people; for instance, being on a sugar high and incapable of regulating one’s own feelings may impinge on a person’s capacity to attentionally focus on a conversation with someone else. Accordingly, to get the full picture of how the affective dimension interrelates with the social dimension of experience always requires also considering the other experiential dimensions that are integrated into and intertwined with a person’s unified experience of the world.

Phenomenological psychopathology is often supported by qualitative investigations that help to elucidate an existential condition through first-person reports and case studies (Køster and Fernandez, 2021). While first-person qualitative material can be helpful for identifying and illustrating the recurring issues and characteristics of an existential condition, in this paper I will focus on such affective phenomena that have already been identified and described in the clinical literature on BPD. Moreover, the aim here is not to reconstruct the whole lifeworld of a given person but to identify possible implications that different aspects of affective disorders in BPD might have for how they experience other people. The possible phenomenological connections I will describe to further illustrate and support the results of my analysis could then be taken as the focus in semi-structured qualitative interviews in the future, phenomenological interviews in particular (Martiny et al., 2021).

## Borderline Personality Disorder as an Existential Condition

Examining BPD through the lens of phenomenological psychopathology means taking BPD as an existential condition. This has two important implications that are easily overlooked. First, when talking about BPD or the condition of BPD, what is meant is a specific *style of experiencing the world*, one that is typically associated with a diagnosis of borderline personality disorder. However, in addressing the phenomenology of BPD, no claim is being made about the concept of BPD that corresponds to the diagnosis or to the criteria that are currently taken to be decisive for the diagnosis to apply. In fact, although the task is to determine the phenomenology of BPD, the phenomenological descriptions presented here do not depend on the recognition of the concept, or on whether a clinical diagnosis has been formulated in a given case or not. That means that a person may find herself in the existential condition of BPD without receiving a diagnosis, or may have received a diagnosis in the past but does not currently suffer from the borderline condition (e.g., during a non-acute phase or remission). Moreover, a person may suffer from aspects of the borderline condition as they are described here (e.g., affective and social experience) without meeting all the criteria for a clinical diagnosis of BPD. Instead, phenomenological claims about the borderline condition and clinical diagnosis are of the following kind: the experience of other people by those who meet the criteria and receive a clinical diagnosis of BPD typically involves the aspects and styles described in what follows. However, this does not mean that these aspects and styles are necessarily specific to BPD, and their description does not by itself serve for purposes of differential diagnosis. Other psychopathological conditions involving comparable affective disturbances may involve similar interpersonal experiences which will be identified as ensuing from affective experiences and how they are processed.

It should be noted that borderline as an existential condition—or the “borderline condition”—is only a *tentative* notion in that it is not yet fully phenomenologically determined. It is not yet clear how exactly the phenomenology of borderline is best described and how phenomenologically different aspects of such an existential condition are connected. It is precisely the task of this paper to provide a contribution to such a phenomenological determination. In this sense, the borderline condition is the explanandum: it is the *target* notion of the phenomenological inquiry that follows.

## Phenomenological Approaches to Borderline Personality Disorder

Phenomenological psychopathology has a tradition that goes back almost as far as phenomenological philosophy. Authors such as Alexander Pfänder (1870–1941) and Eugène Minkowski (1885–1972) shed light on psychopathological disorders through the lens of phenomenological methodology and theorems already in the time of Edmund Husserl (1859–1938). The main focus of these thinkers was aberrant experiential structures in the context of depression and schizophrenia, which continue to be the most examined psychopathological conditions in phenomenological psychopathology. Today, investigations of the experiential structures and their modification have been extended to conditions such as autism spectrum disorders, anxiety, phobias, trauma, eating disorders, and many others, including BPD. However, the experiential conditions associated with borderline, especially as measured against its prevalence and the great turmoil and suffering it causes in persons afflicted with it and in their environment, has received comparatively little attention from phenomenological inquiry. One reason for this is probably the fact that the concept of the disorder is relatively young, tracing back to Adolph Stern’s (1938) introduction of the notion of the *borderline* as referring to patients nosologically located between psychosis and neurosis, and Otto Kernberg’s (1975) term *borderline personality organization*. Moreover, these authors operated within the confines of psychoanalytical theory, which traditionally is considered to be opposed to the phenomenological paradigm, in spite of the fact that there had always been exchange between individual thinkers and researchers working in the two paradigms. It is likely that since BPD was originally a psychoanalytic concept, it did not lend itself to phenomenological investigations, given the prevailing institutional segregation.

In the past decade or so, however, an increasing number of phenomenologically inspired accounts of the different aspects of the phenomenology of borderline have been proposed. Fuchs (2007) has interpreted borderline as a disorder of narrative processes that is closely connected to instabilities in affect, self, and intersubjectivity. Since then, various researchers (Køster, 2017; Bortolan, 2020; Schmidt and Fuchs, 2021) have addressed narrative processes, especially as regards their function of providing a stable sense of self, and how they break down in borderline. Others have focused on the specific way persons with borderline experience time and their heightened focus on the now (Lo Monte and Englebert, 2018), their own body (Køster, 2017), and themselves (Schmidt, 2021a). Aspects of affective experiences have also been investigated. Stanghellini and Rosfort (2013), for example, have identified a specific kind of depression in borderline that is characterized by an oscillation, or “dialectic between dysphoria and anger” (Stanghellini and Mancini, 2018, 3). Another central topos of phenomenological inquiry is the connection between feeling and identity, demonstrating the importance of affect for self-experience (Stanghellini and Rosfort, 2010; Zandersen and Parnas, 2019; Schmidt, 2020, 2021a). Interpersonal issues have been thematized as well. It has been pointed out that emotional experiences in borderline severely distort processes of meaning constitution in a way that disrupts one’s relationships with others (Ferrarello, 2021). Ratcliffe and Bortolan (2021) argue that emotion dysregulation, which is central to borderline, must be considered in interpersonal terms, given that typically self-regulation is socially extended. On this view, it is precisely the chronic interpersonal conflicts typical of borderline that undermine a person’s capacity for emotion regulation and stable emotional experience. Moreover, difficulties with strong and rapidly shifting emotions have been described as undermining the formation and maintenance of a social attentional field, which not only is necessary for an awareness of one’s own emotional feelings vis-à-vis those of others (Schmidt, 2021a) but is also needed for “attaining a sense of a familiarized safe world” (Bader, 2020).

Expanding on these findings about the phenomenological aspects of borderline and their interplay, I will focus here on how disorders in affective experiencing translate into aspects and styles of interpersonal experience.

## Affective Phenomena and Styles of Interpersonal Experience

Some of the phenomena constituting the affective aspects of BPD are more structural (e.g., alexithymia, styles in empathetic processes, and emotion dysregulation) than others (e.g., emptiness and mental pain). All of them, however, as I will suggest in the descriptions of each of them, have an effect on the quality of a person’s experience of the world and especially of other people. I will conclude that these phenomena in conjunction, and the kind of experiential structure they form, may explain a recurring problem for those suffering under the borderline condition: while they feel that they need attachment and connection with others, their actions and behavior often undermine the stability of their relationships.

### Alexithymia as a Disturbance of Interaffectivity

One of the characteristic and core features of the borderline condition is affective instability. This manifests itself in changing moods and emotions and in rapidly emerging and intense affects that are hard to control and often evoke dysregulative behaviors (Nica and Links, 2009). A central aspect of affective instability in borderline is alexithymia, that is, the inability to recognize, identify, and label one’s own emotions (New et al., 2012). Being unable to grasp and express one’s own feelings involves great suffering in itself: As one patient said, “I hate when I am asked what is wrong and I cannot find the words to articulate the unbearable pain I feel inside” (Edwards, 2016, p. 35). But as the quotation indicates, this suffering also translates into interpersonal tension, which stems from the disruption of interpersonal affective attunement, or *interaffectivity*, as described by Fuchs (2013), that is, the continuous process of affective convergence, exchange, and organization of a shared emotional space through emotional communication at basic levels such as facial expression, vocalization, gestures, postures, etc.

What does a lack of or difficulties in affective self-understanding, as it is often prevalent in persons with borderline, mean for interaffectivity? If a person is not able to communicate and resonate with others in a way that would allow them to enter into an emotional exchange, their possibilities for connections with others are significantly reduced (Wastell and Booth, 2003). Instead of an interaffective encounter in which persons can familiarize themselves with the emotions of others, their own strong emotions often lead them into confrontations with others, and even when emotional upheaval does not engender stronger conflicts it can produce an excruciating feeling of isolation. The following case report by Meredith F. Luyten, a therapist describing her experience with her patient Dinah, vividly indicates the need for successful interaffectivity:

“You remind me of my mother, my sister, and John. They’re always after me to express my *feelings*,” she said once, giving the last word an intrusive, insinuating slur. […] Finally […] I told Dinah that I felt bewildered, totally confused as to what she wanted or what I should do. […] Instantly she became tearful, angry […]. I asked her what the tears were about. “I don’t know,” she said with genuine frustration. For the first time I felt Dinah’s panic, and heard her “I don’t know” as a very basic communication. “Are you crying,” I asked, “because of how it feels, how it has always felt, to be pursued, expected to know how you feel, when you just don’t?” She nodded with her head down, and wept quietly. I felt she was ashamed. For the first time we sat together with some relaxation, joined together by the frustration of *not knowing* […]. In this case, the discomfort of not knowing, of confusion and uncertainty, was my exchange with Dinah (Luyten, 1985, pp. 57–58).

As this example shows, failure of processes of interaffectivity can often leave people feeling misunderstood and alone. In turn, interaffectivity can be successful only when a person feels understood to at least a minimal degree, but this presupposes that the person herself has at least a minimal understanding of her own feelings. For only if one grasps at least a bit of one’s own emotions can one possibly feel met by the other’s understanding. Suppose you are in a state of extreme anger, although you are not certain what exactly is upsetting you. While you are aware of yourself as being angry, you do not fully understand yourself or “what this is all about.” Meeting another person involved in the situation, who suggests in conversation that you are angry because of *x* and/or reacts on that assumption, will likely make you feel misunderstood and even isolated. This effect is even stronger when the emotion is more complex than anger. Consider, for instance, an emotional state that involves moments of anger, disappointment, grief, irritation, envy, jealousy, vanity, etc. While you are aware of yourself as being in an uncomfortable emotional state, you do not understand what exactly is the emotion you are experiencing; it is thus even more difficult to convey a sense of one’s own feelings to others, and suggestions and actions by others are less likely to lead to a feeling of being understood. Contrast this with the case in which you are aware of yourself as being angry and disappointed about another person having done *p* or failed to do *q*. They approach you, and even without any need for a long conversation, they do *r* in order to compensate for *p* or having failed to do *q*, in an attempt to allay your anger and disappointment. This will likely produce in you a feeling of being understood, as you can identify *r* as a reaction to your anger and disappointment. You see and experience the other recognizing and acknowledging your emotional feeling and what motivated it. Such an experience of the other as responding to your anger and disappointment involves awareness of oneself as being angry and disappointed. The problem with alexithymia and difficulties in affective self-understanding is precisely that it prevents such a self-awareness, and thus precludes the experience of the other as responsive to one’s feelings in the right way.

The example also illustrates another important point. To identify *r* as a response to your anger and disappointment requires that you be aware of the feelings of the other person, at least to some degree. If you conceive of *r* as an attempt at compensation, this is because you think the other person really understands your anger and disappointment, or at least feels sorry for having caused it. However, alexithymia is not simply a lack of affective self-understanding; it is the more general inability to identify emotions, which also applies to the emotional feelings of the other. That is, alexithymia impinges on empathic processes, which are crucial for successful emotional exchanges.

### The Empathy Paradox, Emotional Contagion, and the Fuzziness of the I-Thou Boundary

Persons with borderline often also show peculiarities in empathy, which not only further undermine processes of interaffectivity but can also have significant implications for their experience of themselves and, not surprisingly, of others. Importantly, findings in the empirical literature are inconsistent: persons with borderline sometimes have been found to be endowed with heightened empathic skills, but sometimes seem to show weakened capacities for empathy (Salgado et al., 2020). Interestingly, even a heightened capacity for empathy appears not to help persons with borderline in preventing dysfunction in their relationships; this has been described as the *borderline empathy paradox* (Dinsdale and Crespi, 2013). One explanation for the paradox makes use of the distinction between cognitive and affective empathy (Harari et al., 2010). Since persons with borderline have difficulty in cognitively grasping and recognizing the emotions of others, they become highly attentive to how others feel and the corresponding emotional cues. But by hyperfocusing on others’ emotions, rather than developing a cognitive understanding of them, they instead develop a heightened disposition toward emotional contagion and reactivity to the affective processes of others (New et al., 2012; Jeung and Herpertz, 2014; Niedtfeld, 2017). Hence, they often demonstrate increased affective empathy but lack skills of cognitive empathy. This is not surprising but reflects their disposition to alexithymia.

#### Structural Changes in Empathically Relating to Others

From an experiential point of view, these characteristics in the borderline style of empathy can be interpreted as an expression of a specific style of experiencing other people and their emotions. A person who has difficulties in recognizing their own emotions as well as those of others experiences the process of interaffectivity in a very particular and intense way:

Exchange was terrifyingly real for Dinah. […] A shared feeling state could be experienced as a powerful extension of self, or as loss of self, as an opportunity for manipulation or loss of control, as a flood of information or as sudden disorientation (Luyten, 1985, p. 58).

A further problem with not being able to identify one’s own and others’ emotions is that it also becomes difficult for the person to *distinguish* between one’s own feelings and those of the other. Max Scheler’s distinction between empathy (*Einfühlung*) and the feeling of oneness (*Einsfühlung*) may help to illustrate this (Bolley, 1964). The feeling of oneness is characterized by a collapse of the I with another consciousness. Instead of experiencing one’s own feelings vis-à-vis the feelings of the other, persons with borderline tend to find themselves drawn into an all-encompassing affective field that lacks a more pronounced articulation. For instance, the (rightly or wrongly) perceived boredom of a close friend may directly translate into fear of loss. The other’s boredom and one’s own fear of not being entertaining enough merge into a single, diffuse process of fear of loss, involving both boredom and insecurity as inextricably intertwined moments. Instead of having an awareness of two distinct emotional processes that could enter into an interaffective exchange that would constitute an encounter with another person, a person with borderline tends to live through what is a form of affective fusion that undermines the I-thou boundary (cf. Schmidt, 2021a). When the border between self and other is fuzzy, such a fusion can be experienced in different ways. It can trigger feelings of anonymous loneliness in which both self and other seem to disappear, but it can also result in a feeling of “claustrophobia” (Luyten, 1985, p. 49) in which one feels consumed by the other, as it were.

Though typically associated with suffering, fusion can also be experienced as the ideal state of love and connection, evoking desires of perfect harmony, an ideal that can hardly be met by any relationship and thus will frequently be followed by painful and traumatizing disappointment. In fact, the tendency of persons with borderline to seek relationships that consist in a merging with the other bereft of any friction can, I suggest, be interpreted as a mode or aspect of a general diminishment of the I-thou boundary that results from a lack of affective understanding. Lacking positive experiences of and capabilities for successful interaffective encounters in which two individuals can experience harmony as a *process* of alternating phases of synchronization and desynchronization, persons with borderline seem to seek a perfect harmony as a *state* that is never at risk. The ideal of a partner in relationships then is not one with whom a continuous process of interaffective synchronization is possible but one with whom a state of fusion can be attained. The ideal of fusion can be manifest in the borderline-typical ambivalence, intolerance, or hypersensitivity to cues that may indicate changes in relationships and hyperreactivity to anything short of fusion (Frick et al., 2012).

#### Manipulative Behavior as an Expression of Inhibited Interaffectivity

A lack of empathic ability can also lead to the emergence of maladaptive strategies for better grasping what others are feeling and thinking. The struggle for perfect harmony and attachment often also entails a heightened felt need to recognize the other’s feelings in order to be able to monitor the current degree of fusion. For interpersonal fusion often seems to function as a general prototype for borderline relationships, motivating an increased need for individuals to know how others feel and why exactly. However, since the process of interaffective exchange is disturbed, persons with borderline are typically seen as developing alternative strategies for emotional transactions and synchronization that are often labeled manipulation. Without entering into the important and controversial debate whether such a label is adequate (see Potter, 2009, Ch. 6) and taking “manipulation” here only as a placeholder in the absence of a better term for the borderline strategies in question, I want to suggest that these behaviors can be regarded as maladaptive and compensatory attempts to overcome what are perceived deficits in interaffectivity and lack of successful exchange with others (cf. Schmidt, 2021b). In this sense, they may be seen as behavioral correlates expressive of the fuzziness of the I-Thou boundary and of experiences of fusion in its different modes.

For instance, manipulation can serve an epistemological purpose (Stanghellini, 2014). Eliciting strong emotions and clearer behaviors in others through provocation can make it easier for the person with borderline to understand how others are feeling and so reduce the insecurities that stem from ambivalence. In this sense, manipulation seems also to be used as a tool to test the quality of a relationship, as Stanghellini’s report of the interaction with one of his patients illustrates:

During the therapy sessions she sits restlessly, remains silent and answers my questions in a provocative way. During one of the following sessions she will explain that she needed to test my interest in her, if I really cared about her, and my intention and capacity to understand her in her moody days (Stanghellini, 2014, 13).

Adding to and further developing Stanghellini’s approach, I wish to suggest that manipulation can also have the following functions:^1^

• *Affective self-understanding*: Transferring one’s own emotions to others by manipulation can also turn out to be helpful for better grasping one’s own emotions, in that the behaviors of others may be more palpable than one’s own feelings.
• *Emotional communication*: Given the condition of alexithymia, manipulation can also be seen as a form of emotional communication that allows a person to express their own feelings by eliciting them in others. Manipulation in this sense might be considered the counterpart of the influx of emotional contagion that persons with borderline often live through; that is, it is the other side of affective processes that seem to lack clear boundaries.
• *Liberation from claustrophobia*: When a person feels claustrophobic in a relationship, inducing emotional feeling in the other may provide release, be it by dominating the other’s affect and the amalgamated affective field, or by creating interpersonal conflicts that may dissolve the state of fusion.
• *Establishing connection*: In other cases, manipulation can be regarded as a way of creating a state of affective fusion with another person. For manipulative behavior not only can have a synchronizing effect by transferring one’s own feeling to others, it may also increase the attention and reactivity of the other toward the person with borderline. Manipulation will thus link the emotional processes of the manipulated with those of the manipulator. Even if the manipulated individual does not share the exact same feeling as the manipulator, the irritation that manipulative behavior can induce may establish in the manipulated individual the same kind of hypersensitivity to subtle emotional cues. For instance, the manipulated individual might devote more of their attention to the feelings and motivations in the manipulator that might lie behind their attempts at manipulation. This may establish a shared tension that can present the prototype of an affective field in which the experience of both individuals collapses more and more into one seemingly dissolvable and shared emotional atmosphere of hypervigilance and irascibility, which is often the entry into a relationship without boundaries.

To sum up, rather than considering manipulation a malignant trait that adds to the stigmatization of patients with borderline (Ring and Lawn, 2019), I propose to see it as the expression of a particular style in the structuring and processing of emotions and in the experience of others (Schmidt, 2021b). This style, I suggest, results from the difficulties in affective understanding and the weak I-thou boundary. On this view, manipulation and the gravitation toward fusion are both the surrogates for and residua of a hindered interaffective exchange that normally strives toward synchronization.

### Emotion Dysregulation: The Role of Others When Things Get Out of Control

Another important aspect of borderline affective instability is a low capacity for emotional regulation, which is also associated with heightened impulsivity (Henry et al., 2001). Dysregulative emotional processes can be partially explained by what I have described as a lack of affective understanding and the resulting disturbed interaffectivity. For one, without being able to properly identify and communicate emotions in oneself and others, it is obviously much more difficult to mitigate and control emerging impulses and emotional feelings (Schmidt, 2020, 2021a). Moreover, many of our means of dealing with painful and challenging events involve other people, as regulatory processes are in many cases socially distributed, such as talking to a friend or doing sports (Varga and Krueger, 2013). Given the disturbance in interaffective processes and the concomitant recurring interpersonal calamities, interpersonally structured forms of regulation are often not available to persons with borderline, further decreasing the repertoire of regulatory means available to stabilize one’s own affect (see Ratcliffe and Bortolan, 2021).

#### Lack of Control Is an Experience

Apart from these important aspects involving a general intertwining of emotional and interpersonal processes, I want to suggest that a lack of regulatory capacities also has significant repercussions for how a person experiences others. On this view, emotion dysregulation is a phenomenon that prefigures the way we relate to partners, friends, family, colleagues, and strangers, and how interactions with them are experienced. To understand this, the first thing to note is that the lack of adequate regulatory skills inscribes itself at an experiential level. It feels somehow like being unable to control one’s own emotions, as the following first-person report reflects:

My emotions are escaping me. They are becoming more numb and dull. I don’t have much control over anything anymore, as if my body is on auto-pilot…. Headed for a mountain (Edwards, 2015, 127).

Statements like this show the level of exposure and vulnerability that result from lacking regulatory skills. This is an important point to note, since the general perception is that in interpersonal conflicts it is the person with borderline who has shown provocative or hurtful behavior; that is, they are the one who is the epicenter of the turmoil. Crucially, even in situations where this might be the case, the active behavior of the person with borderline has structurally a strong passive experiential character in that emotions and impulses are perceived as overwhelming, which is what drives them to inappropriate and dramatic behavior in the first place. These circumstances typically create a huge gap between the person with borderline and their social environment, leaving them isolated and chronically misunderstood.

#### Emotion Dysregulation and Three Typical Roles of Self and Other

Given that lack of control manifests itself at the experiential level, it is not surprising that shame, guilt, and blame are also central in the experience of BPD (Peters and Geiger, 2016). Obviously, such issues cannot be separated from the experience of other people: dealing with different experiences of loss of control—together with shame, guilt, and blame—implies a certain style in relating to others. Stanghellini and Mancini (2018) observe that relationships of borderline persons often have a traumatic character; as a result relationships are often structured according to the roles that figure in the experience of a traumatic event. In this sense, the borderline person experiences herself as a *victim*, a *perpetrator*, or a *bystander* (Stanghellini and Mancini, 2018, 14–15). All these roles involve a certain experience of oneself as passive and responsive, for even in the case of experiencing oneself as a perpetrator, the person with borderline experiences her own behavior as a “sort of reflex, an automatic response she simply could not control” (Stanghellini and Mancini, 2018, 15).

What I want to suggest is first, that the three roles that determine typical relationships with others present a pre-structured space of possibilities in which the other can be experienced, and secondly and more importantly, that this interpersonal space of possible relationships is an almost direct phenomenological implication of the lack of regulatory capacities. More specifically, I contend that taking the stance of a victim, perpetrator, or bystander are different attempts to grasp the fundamental passive experience that is associated with a lack of regulatory skills. Crucially, persons with borderline typically do not and need not limit themselves to only one of these roles; rather, what seems to be often the case is an overwhelming and all the more confusing shifting among the three roles. The following quotations, from three different entries posted by Topher Edwards on the same day in his online diary, could be interpreted as expressions of such an oscillation:

(1) Looking back on my life, I realize that I have a lot of blood on my hands. Some is mine, some of the girls’ whose hearts I ripped out […]. […] Run motherfuckers, the smile isn’t real.
(2) So I throw up my middle finger in rage and scream FUCK YOU! to all those who have left me over the last few years. […] This isn’t all my fault, motherfuckers. YOU did this too.
(3) (D)ay after day, I sit alone… isolated. No one to visit, not even anyone to call or text. This is getting really FUCKING old. What is it about me that seems to repel people? Do I give off a scent of negativity, prompting everyone to run? (Edwards, 2015, pp. 119–120)

In the first quote, feelings of being a perpetrator may dominate, whereas in the second Edwards seems to find himself in the role of the victim. The third quote is not related to specific others; rather, Edwards here seems to reflect on the general relationship between himself and others, which can be regarded as a case in which a person experiences himself as a passive bystander of an inevitable process that intimately concerns oneself and one’s own characteristics but which lies beyond one’s own control.

Such oscillation not only has a debilitating effect on the already weakened sense of self in persons with borderline, it also further isolates them from others and solidifies a feeling of disconnectedness: “When I think of relationships, I think of pain, heartbreak, self-destruction, and the feeling of being completely alone. […] In my mind, no one deserves the pain I can cause” (Edwards, 2015, p. 88). While being a perpetrator and victim may still present strong forms of connectedness to concrete others and involve meaningful relationships, taking on the role of a passive bystander of a more general process implies a distancing both from oneself and from others. Being forced by the grueling oscillation between accusation and self-blame into the position of a powerless spectator, the borderline person will often find herself emotionally drained and puzzled. Edwards describes this on the day of his three posts: “I cannot make sense of my life. I’ve used many words to describe it, but the only one that seems to fit is ‘empty”’ (Edwards, 2015, p. 120). Thus, the oscillation among roles attributed to oneself and others culminates in a general disconnectedness: self-alienation as well as disruption of contact with others, a circumstance that is present in strong feelings of emptiness.

However, feelings of emptiness, another core moment in affective instability in borderline, should not be seen only as a possible end point of emotion dysregulation and role diffusion. Rather, as a prevailing condition in borderline, emptiness itself has phenomenological implications, in that it gives rise to peculiarities in the way a person experiences others.

### Emptiness as a Social Phenomenon

Without claiming to deliver a profound and sufficient discussion of the complex phenomenon of emptiness, I want to draw attention to a few aspects of feelings of emptiness that are relevant for interpersonal experience. Before doing so, however, I wish to point out that the feeling of emptiness, although pervasive in borderline and a chronic condition, is not a universal phenomenon. Feeling empty, apart from the fact that it is typically associated with an unbearable “mental pain” (Tossani, 2013; Fertuck et al., 2016), can feel quite different in different contexts (Køster, 2021, pp. 127–128). In this sense, which some might strike as surprising, emptiness is not that different from what might be considered the opposite of emptiness: feeling overloaded and overwhelmed by an excess of meaning. Feeling flooded by experiences of significance and experiencing events as highly meaningful surely has some general aspects, but how exactly it is phenomenologically experienced usually depends on the context. The same, I suggest, also applies to the absence of significance that characterizes emptiness. How emptiness is felt depends on the context in which a person feels empty. Bearing this in mind, the following descriptions are intended only as two *examples* of how emptiness can translate into an impaired social experience; they are not meant as anything approximating a comprehensive characterization of the phenomenon of emptiness (cf. Zandersen and Parnas, 2019).

#### Masquerade and the False Self

Zandersen and Parnas (2019) report a case in which a patient describes her emptiness as a “sort of painful restlessness and anxiety without autonomic symptoms,” a condition in which she feels “her ‘I’ as being so blurry that she sometimes thinks she cannot even die because there is no ‘core’ that can be ‘taken out of the game”’ (p. 111). Edwards (2015) feels “like a ghost” (p. 45) and that he is “only just existing” (p. 49).

Independently of why and how such feelings arise (which would itself require a detailed analysis), what do they mean for how others are experienced? Crucially, such feelings typically lead to the weakened sense of identity that is characteristic of borderline (Fuchs, 2007; Schmidt, 2020, 2021a; Kaufman and Meddaoui, 2021). For without a sufficiently strong feeling of self, any roles or identities that are constituted in various life contexts, such as being a friend to *p* or a parent of *q*, will feel wrong, artificial, or inauthentic. Accordingly, the empty person will feel the need to develop what Jørgensen has called a “false self” (Jørgensen, 2006, p. 635) in order to function in the social world. Again, Edwards’s words are illuminating: “To be honest, I am growing tired of this masquerade. […] I go through life doing what needs to be done to fulfill my role in society” (Edwards, 2015, p. 49). It is no wonder that a person who feels empty in this way may develop a condition in which “she [can] no longer fundamentally believe in the reliability of any identity” (Luyten, 1985, p. 49). Feeling empty amounts to a mismatch between how one experiences oneself and the identities and roles that seem to have accrued to oneself. Not only is such a mismatch painful and tiring, it also exacerbates the experience of isolation. For it is not just that interaffective exchange with others is hindered by one’s lack of affective understanding and regulative capacities; additionally, if one does not feel oneself to be this or that person with these individual character traits, then it is also very difficult to have an experience of a real encounter with others. If one feels that one is always wearing a mask that conceals one’s own being, no real contact with another person is possible.

Moreover, distrusting one’s own identity and role in social contexts will also likely undermine trust in others. Feeling empty extends beyond self-experience, for it is a condition in which nothing seems significant, enticing, or meaningful. Accordingly, I suggest, this applies also to how other people, in their behavior and communication, appear to a person suffering from the borderline condition. Feeling empty motivates a general suspicion toward anything that claims meaning, and so implies a crisis of authenticity. Emptiness might thus explain the fundamental need for authenticity that has been attributed to persons with borderline (Stanghellini and Mancini, 2018), a need that at the same time cannot be fulfilled by any other person, since whatever the other does will typically be interpreted by the borderline as just a further instance of inauthenticity, like a play of masquerade. The behavior of others is seen as concealing the real intentions behind it. To be able to overcome the masquerade, the borderline would have to expose herself in a sincere way by showing what she thinks and feels about things. However, feeling generally empty, and so lacking a good grasp of one’s own emotions, this is hardly possible.

#### Emptiness Amounts to Disconnection, Insecurity, and Fear of Loss

Emptiness, regardless of a person’s emotional and social skills, is a feeling that is hard to communicate, and it lends itself to sharing with others only in a very limited way. Even where there is a mutual recognition of emptiness in the other, this does not lead to a strong sense of emotional sharing, compared, for instance, with that involved in enjoying a shared activity such as watching a movie together. Emotional sharing that constitutes a connection between the emotional processes of two individuals and involves a form of we-intentionality is more than the fact that two people undergo the same emotional experience and recognize it in the other (Salice, 2022); it involves a “feeling of being *together* in this.” Many emotional experiences, such as anger, joy, hate, or attraction, allow for such moments of sharing; emptiness, by contrast, has a strong disconnecting aspect, so that even when it is shared by two or more people, it does not give rise to a we-experience in the relevant sense.

Accordingly, feelings of emptiness not only are not easily communicated, but when chronic and recurring they also significantly undermine a person’s general ability to share emotions with others and thus to make relevant experiences of emotional togetherness. This can have a severe effect on the experience of interpersonal relationships. Imagine, for instance, that you are having dinner with a person you love. Both of you are enjoying the meal, the cheese, the wine, etc. During your (admittedly rather minimal and superficial) conversation, you feel relaxed. Everything is fine and you feel secure with your partner. Likely this will come with an experience of stability of relationship, even if you have not discussed or assured yourself that the other person feels the same way. By contrast, imagine you are sitting at dinner and you feel emotionally drained and empty. Meal, cheese, and wine are fine, but they don’t exactly lift your spirits. Your mood has faded, and now the conversation seems unnecessary and meaningless, and you aren’t particularly enjoying the setting, despite the candles your partner has put on the table. Something is missing but you don’t have any idea of what it could be.

What I want to suggest is that such a feeling of emptiness amounts to a discomforting experience of insecurity. In the borderline, this typically motivates a destructive process of inquiry that can consume every aspect of relationship: Is the other person perhaps not as interesting as I had thought? Are they also bored? Am I boring or unattractive? Should I do something about it? Is this what a relationship should feel like? Are we meant to be together? Does this person doubt our relationship? And so on. Thus, in the absence of the connecting experience of shared emotions, emptiness almost always triggers a fear of loss, even prior to the maladaptive and detrimental behaviors that often ensue when someone suffering from the borderline condition attempts to manage her relationship with others in order to reconnect.

### Mental Pain and Destructive Behavior: Interaffective Disorder as a Social Impairment

Emptiness is not simply a deprivation—a deprivation of meaning, of self-feeling, of interpersonal connection; it also has itself a qualitative aspect. It is a “nothing that is something” (Korner et al., 2008). In borderline, emptiness in conjunction with feelings of loss of control and a sense of woundedness, loneliness, and helplessness, results in an “unbearable mental pain” (Fertuck et al., 2016, 2), which has also been characterized as a “desperate vitality” (Stanghellini and Rosfort, 2013). Some have emphasized that mental pain—also variously referred to as dysphoria, emotional pain, or psychic pain—is a residuum of (traumatic) experiences of early attachment (Korner et al., 2008). Others have suggested that it is rather what fuels the recurring interpersonal conflicts and keeps them going (Zanarini et al., 1998; Pazzagli and Monti, 2000). From an experiential point of view, there is no need to decide on a single explanatory direction. Likely, painful interpersonal experiences in early infancy have a significant impact on how a person learns to process emotional feelings, thus pre-structuring how affective experiences are generally lived through. Studies on the effect of unsupportive parental environments corroborate that view (Grove and Crowell, 2019). On the other hand, the phenomenology of chronic mental pain will plausibly have an impact on how a person perceives others. How then does mental pain in borderline influence the structuring of interpersonal experience?

To answer this question, more needs to be said about the phenomenology of mental pain. Crucially, while one should not forget that a person will suffer their own pain within their own individual life context, there are a few structural aspects that are common to the experience of mental pain independent of its contents (i.e., what individual mental pain is about). For one, although mental pain is precisely not reducible to bodily pain, it does have a strong bodily aspect. People feeling mental pain typically find themselves in states of high bodily tension or chronic autonomic arousal (Williams et al., 2001), accompanying not only a sense of lacking control and a fear of loss, but mostly a sense of rejection and felt self-devaluation. In fact, while mental pain is not specific to borderline, what does seem to be specific to mental pain in borderline is the great importance of feelings of worthlessness and a proneness to feeling humiliated (Fertuck et al., 2016). For instance, as a result of past failed relationships, the emotional pain resulting from loss can translate into more habitualized ways of feeling worthless, as another quote from Edwards illustrates: “When people I cared about, and thought cared about me, left me, it was a huge shot to my self-worth” (Edwards, 2015, 6). The point here is that the resulting “purgatory, endless pain” (Edwards, 2015, p. 25) itself has an effect on interpersonal experiences to come: “Your self-esteem can become so low that it is practically non-existent. It is a vicious cycle brought on by not only how you view yourself, but by your idea of how others perceive you” (Edwards, 2015, p. 6). Each new relationship that could help to overcome emotional injuries is already burdened by excruciating pain; this, I suggest, underlies statements such as “Reparation seems like an impossible dream” (Edwards, 2015, p. 63) and “There is too much pain […] to be able to move on and let go” (Edwards, 2015, p. 57).

Such pain and the disruption of felt self-worth evidently shapes how a person sees others. Edwards notes: “My usual way of coping is to take it out on myself. […] I’d rather cut myself than someone else” (Edwards, 2015, p. 6). Mental pain, I suggest, not only is directed at those who might have been involved in a painful interpersonal conflict, but crystallizes into a general experiential attitude toward others. This becomes evident in Edwards’s case when he emphasizes the general vulnerability attached to friendship: “When you give yourself to someone else, you hand them the ability to destroy you as well” (Edwards, 2015, p. 11); “I define friendship as giving someone a knife and expecting them not to stab it deep into your back” (Edwards, 2015, p. 60). Rather than being a source of security and connection, others are experienced as a threat, a source for just more pain and experiences of loss or what is often, as in Edwards’s case, perceived as betrayal.

It is easy to see how mental pain, and the irascibility and fundamental sense of vulnerability that come with it, can influence social encounters and how they are experienced. What is more puzzling is why people with borderline display the kind of behavior they do, which is ultimately self-destructive and detrimental to their relationships: “Relationships? Blah. I will only destroy them. But at the same time I feel it is what I need” (Edwards, 2015, p. 67). Edwards asks himself, “Why do I need to fuck shit up?” (Edwards, 2015, p. 123), fully aware of the paradox that characterizes the borderline condition: although desperately in need of others and of interpersonal connection, persons with borderline often initiate or even welcome the destruction of their relationships, or at least contribute to it.

Without aiming at a reductive explanation, I want to suggest that we can make sense of this paradox by looking not only at the quality of chronic mental pain that forms the background of the borderline condition but also, and in conjunction with it, at the structure of affective experiences as I have described it throughout the paper. On this proposal, the different affective phenomena, taken together, may not only shape how a person with borderline experiences other people but also provide the motivational background of certain actions and behaviors in the context of interpersonal conflicts. I have argued that alexithymia, the lack of affective self-understanding, borderline-related styles in empathy, emotion dysregulation, and chronic emptiness significantly undermine emotional exchange. Individuals with borderline struggle with processes of interaffectivity, the I-thou boundary can become blurry, and a general sense of estrangement from others arises, which often manifests as a chronic mental pain involving feelings of rejection and worthlessness. Under these emotional conditions, especially that of mental pain, another difficulty arises for those suffering. It is this difficulty that is, in my view, what triggers destructive actions and behaviors in interpersonal contexts, but is also among the factors with the most long-lasting effect with regard to the maintenance of the borderline condition. What is this problem?

The problem consists in the fact that for someone who is chronically and repeatedly in emotional pain to connect with others means to share their pain with others. However, for someone who is experiencing the complex mental pain individuals with borderline experience, emotional exchange in which a person can feel met and understood by someone who is *not* living through the same kind of emotional pain is seldom an option. In fact, the very foundations for possible emotional connection are generally undermined. Individuals with borderline, although aware of their mental pain, struggle with understanding their pain in its complex and concrete meaning as well as conveying it to others. Hence, they do not feel met emotionally by others. As a result, the typical way for them to communicate their emotions and make themselves understood is, as described above, to produce similar feelings in the relevant other. Thus, the desire to be understood, to *share* one’s own emotional feeling and connect emotionally with the other, translates into a desire to make the other *be* in comparable emotional states. Clearly, with mental pain, this will be a significant problem, as it will typically result in interpersonal conflicts. Though actions and behaviors that aim at producing pain in the relevant other might be meant as attempts at emotional bonding, fusion, and ultimately connection, they are self-defeating, since they must be seen, and mostly are seen as hostile. The conflicts that result from this typically lead to disruptions in the relationship, adding to the mental pain rather than alleviating it.

One way or another, this means that those in chronic mental pain will be left to themselves. Phenomenologically speaking, chronic mental pain, in conjunction with the structural aspects of the disorder of interaffectivity, not only amounts to a disconnection from others, it also triggers actions and behaviors that are detrimental to social relationships. Accordingly, the affective instabilities that make up the disorder of interaffectivity are a significant and painful social impairment.

## Implications for Therapy

How can insights into the affective aspects of interpersonal experience in borderline inform treatment? How can you help someone who is experiencing complex and chronic mental pain, feels cut off from everyone, and has tremendous difficulties in exchanging emotional feelings with others? How do you help someone who is desperate and finds themselves sometimes even needing to make others experience comparable states of pain so as to be able to share emotional feelings with them and feel connected with them?

A first conclusion I draw from my analysis is that those issues that form structural aspects of the whole affective instability complex should be addressed in therapy. This is in line with both Linehan’s Dialectic-Behavioral Therapy (Linehan, 1993; Dimeff et al., 2021) and psychoanalytical approaches (Kernberg, 1975; Bateman and Fonagy, 2010), which focus on improvements in emotional self-regulation and empathetic skills. It also further underscores the therapeutic relevance of feelings of connectedness in the treatment of borderline (Kverme et al., 2019). Given the specific chronic mental pain that people with borderline bring with them into any social relationship of any depth, emotional exchanges between therapist and patient pose significant challenges for the therapist: for instance, because for the borderline patient to feel met means for the therapist to join them in the sphere of emotional pain.

This is illustrated vividly by Luyten’s case study of her patient Dinah. Her inability to make sense of her emotional life, and of life generally, was a point of focus in the therapy. Luyten describes how she had attempted to make several suggestions to Dinah to explain how and why she felt the way she did in certain situations. None of the suggestions were accepted by Dinah, resulting in an insecurity on Luyten’s part: “I felt exhausted, irritable, and on guard. […] I needed to be doing something, but instead I felt panicked by not knowing what was going on. The feeling of *not knowing* was much more disturbing in this therapeutic relationship than in any other which I had undertaken” (Luyten, 1985, p. 57). Eventually, Luyten confronted her patient: “I told Dinah that I felt bewildered, totally confused as to what she wanted or what I should do” (Luyten, 1985, p. 57). Over the course of the therapy, Dinah had managed to evoke in Luyten feelings of emptiness, panic, and frustration about not knowing what to do that were similar to her own feelings. While this might be interpreted as a failed therapy, Luyten emphasizes how important her own emotional process was for the therapeutic relationship: “For the first time we sat together with some relaxation, joined together by the frustration of *not knowing*, and appreciating the shame and fear that evoked” (Luyten, 1985, pp. 57–58).

While this kind of connection might not suffice to alleviate and deal with the whole complex of suffering involved in the borderline condition, it not only has a healing effect itself but also can be the first, necessary step to opening an interaffective space with others in which personal issues can be addressed. Similar ideas have been described in the context of “relational integrative psychotherapy” (Finlay, 2016, Ch. 5.).

To enable a successful emotional exchange between therapist and patient, another insight of the analysis is of key importance: the distinction between *existential condition* and *character trait*. Therapy with borderline patients can be demanding, as it requires the therapist to engage in emotional exchanges involving their own emotional feelings and with their personality as a whole. In therapies involving borderline, more so than in the case of other psychopathologies, the therapist must act and appear as another person, someone who can represent others in a way that allows the patient to learn new ways of experiencing interpersonal interactions. To facilitate such interactions, it is crucial to understand the mental pain and the destructive behaviors that might ensue from it, as an aspect of borderline qua existential condition and its experiential manifestations rather than as a character trait of the patient. On this view, mental pain and the interaffective disorder constitute a social impairment that triggers the desire to have others feel a pain comparable to what one feels oneself, given that it is only under such conditions that the patient can develop a sense of shared emotional feeling and connection. The idea is thus that everybody who suffers from a similar existential condition will develop the longing for others to join the sphere of mental pain. Destructive tendencies are thus seen as a reflex of sorts pertaining to the existential condition. These tendencies are a hindrance to the development of a person’s individual character rather than a part of that development. Bearing this in mind might help therapists to better tolerate and so better deal with conflict-seeking behaviors in their patients. It might also help patients to develop a sense of themselves as distinct from their existential condition, as well as invoke the idea that transcending their existential condition will allow them to enter into more successful relationships with others.

## Conclusion

How do persons with borderline experience others, and how might addressing this question help in understanding and explaining their recurrent interpersonal problems? To address this question and to contribute to a possible answer, I have suggested examining the affective phenomena typically found in borderline from a phenomenological perspective in order to identify how structures of emotional experiencing may translate into ways of relating to others. Taking borderline as an existential condition that consists of a certain configuration of experiential aspects, I have argued that the affective phenomena involved in borderline amount to a disorder of interaffectivity. This disorder, as I have described it, includes alexithymia, decreased cognitive and increased affective empathy, and emotion dysregulation.

As a result of these structural phenomena, the individual has difficulties in experiencing emotional exchange with other people, resulting in blurred I-thou relationships. The fuzziness of the self-other distinction itself destabilizes social relationships and is a source of frantic efforts either to maintain fusion-like forms of attachment or to rid oneself of claustrophobic feelings when fusion becomes unbearable. One way or another, under such structural (inter)affective conditions, a person with borderline is likely to feel disconnected from others or to become isolated due to disruptions of relationships with relevant others.

Associated with this structural affective complex are chronic feelings of emptiness and mental pain. These feelings further complicate matters, for they increase the level of suffering in itself but also because they trigger the desire to make others feel the same kind of pain they do. However, this desire and the behaviors motivated by it conflict with the need for and attempts at interpersonal connection. I have argued that such a paradoxical experience and behavioral style is not to be interpreted as a (negative) character trait but rather as a corollary of the borderline style of experiencing the world, others, and themselves. The paradox arises due to their fusion-like style of relating to others, in which sharing and communicating an emotional feeling requires an almost perfect identity of feeling. In the case of chronic mental pain, this means that a person, in order to feel understood and connected, develops the need to have relevant others accompany them in their affective sphere of mental pain. Thus, while actions and behaviors that may inflict pain upon others are often seen as expressions of hostility, they are often aimed rather at emotional exchange and connection.

Understanding such actions and behaviors as manifestations of an existential condition involving a trouble-generating mode of interaffectivity rather than as a specific feature of an individual’s personality may allow therapist and patient to thematize associated issues in a non-stigmatizing way. At the same time, understanding how certain needs and ways of experiencing others result from the structure of emotional processing may help the patient develop a new perspective on themselves: the idea that changing the way one experiences affect, and so altering one’s own existential condition, may enable new ways of relating to others. While stabilizing affect and interaffective processes alone may not suffice to settle all the issues a person is typically confronted with in their individual borderline condition, it may make possible the first steps to feeling met by and connected with others.

## Author Contributions

The author confirms being the sole contributor of this work and has approved it for publication.

## Conflict of Interest

The author declares that the research was conducted in the absence of any commercial or financial relationships that could be construed as a potential conflict of interest.

## Publisher’s Note

All claims expressed in this article are solely those of the authors and do not necessarily represent those of their affiliated organizations, or those of the publisher, the editors and the reviewers. Any product that may be evaluated in this article, or claim that may be made by its manufacturer, is not guaranteed or endorsed by the publisher.

## Footnotes

1 Related—though far from congruent—ideas about manipulative behavior can be found in psychoanalytic psychology and psychotherapy under the label *projective identification.* However, the term’s scope is much wider than the functions of manipulative behavior I have in mind here; see Roth (2005).
